# Analysis of the Microbial Community Structure in Coastal Sediment of an Ascidian Farm in South Korea through 16S rRNA Gene Amplicon Sequencing

**DOI:** 10.1128/MRA.00584-21

**Published:** 2021-07-29

**Authors:** Ilwon Jeong, Jong-Oh Kim, Seokjin Yoon, Kyunghoi Kim

**Affiliations:** a Department of Ocean Engineering, Pukyong National University, Busan, Republic of Korea; b Institute of Marine Biotechnology, Pukyong National University, Busan, Republic of Korea; c Dokdo Fisheries Research Center, National Institute of Fisheries Science, Pohang, Republic of Korea; University of Southern California

## Abstract

Aquaculture places contamination pressure on the coastal environment. We investigated the microbial community structure changes in sediment in an ascidian Styela clava farm. Data profiling of the 16S rRNA gene amplicon sequence shows that the microbial diversity of sediment in the Styela clava farm is dominated by *Proteobacteria* (relative abundance, 95.34 to 97.85%).

## ANNOUNCEMENT

Ascidian Styela clava farms exist only in Republic of Korea, especially because Styela clava is cultivated only in Jinhae Bay (1). Jindong Bay is in the northwest section of Jinhae Bay and is mainly used as a Styela clava farm. Jindong Bay has a low seawater exchange rate (35% in 100 days) with water current velocity of 3 to 10 cm/s, resulting in high mortality rates for Styela clava (2). Several environmental investigations have been conducted to decrease the mortality rates for Styela clava (1, 3). Although aquaculture has the potential to deteriorate the benthic environment due to the accumulation of pollutants (3), there has been less environmental evaluation related to the benthic environment in the Styela clava farm. Therefore, it is necessary to investigate the effect of the Styela clava farm on the benthic environment. In this study, the microbial community structure changes in sediments from the Styela clava farm were investigated through 16S rRNA gene amplicon sequencing.

Sediment samples were collected from Jindong Bay (35°5.9120′N, 128°28.3750′E) in April, August, October, and December 2019. Using a Peterson grab sampler, surface sediment samples from a depth of 15 cm (water depth, 3 m) were collected in 1-liter sterile high-density polyethylene (HDPE) bottles; the samples were stored immediately at –20°C for DNA analysis (4). The samples were delivered to Macrogen, Inc. (Seoul, Republic of Korea). Total DNA was extracted from 10 g of sediment using the DNeasy PowerMax soil kit (Qiagen) according to the manufacturer’s instructions. The 16S rRNA gene amplicon sequencing libraries were prepared with Herculase II Fusion DNA polymerase and the Nextera XT index kit v2 with the primers Bakt_341F and Bakt_805R according to the manufacturer’s guidelines. Paired-end sequencing was conducted with the Illumina MiSeq platform. The raw data obtained (301 bp long) were assembled with high quality scores (average score, >20) (4, 5). Using an identity cutoff value of 97% similarity, the operational taxonomic units (OTUs) were clustered with QIIME v1.8.0 against the Ribosomal Database Project (RDP) database. A total of 582,367 raw reads and 75,771 OTUs were sequenced for 16S rRNA gene libraries (Table 1). The total OTUs were assigned to 23 bacterial phyla, 48 classes, 85 orders, 148 families, 372 genera, and 527 species.

**TABLE 1:** Summary data obtained for sediment samples from Jindong Bay

Parameter	Data for sample collected in:			
	April	August	October	December
No. of reads	147,232	188,758	140,213	106,164
No. of OTUs	15,819	23,077	18,890	17,985
Proportion of bacteria (%)	97.55	96.36	95.34	97.85
Proportion of archaea (%)	0.01	0.01	0.05	0.01
SRA accession no.	SRX11044005	SRX11044006	SRX11044007	SRX11044008

**FIG 1:**
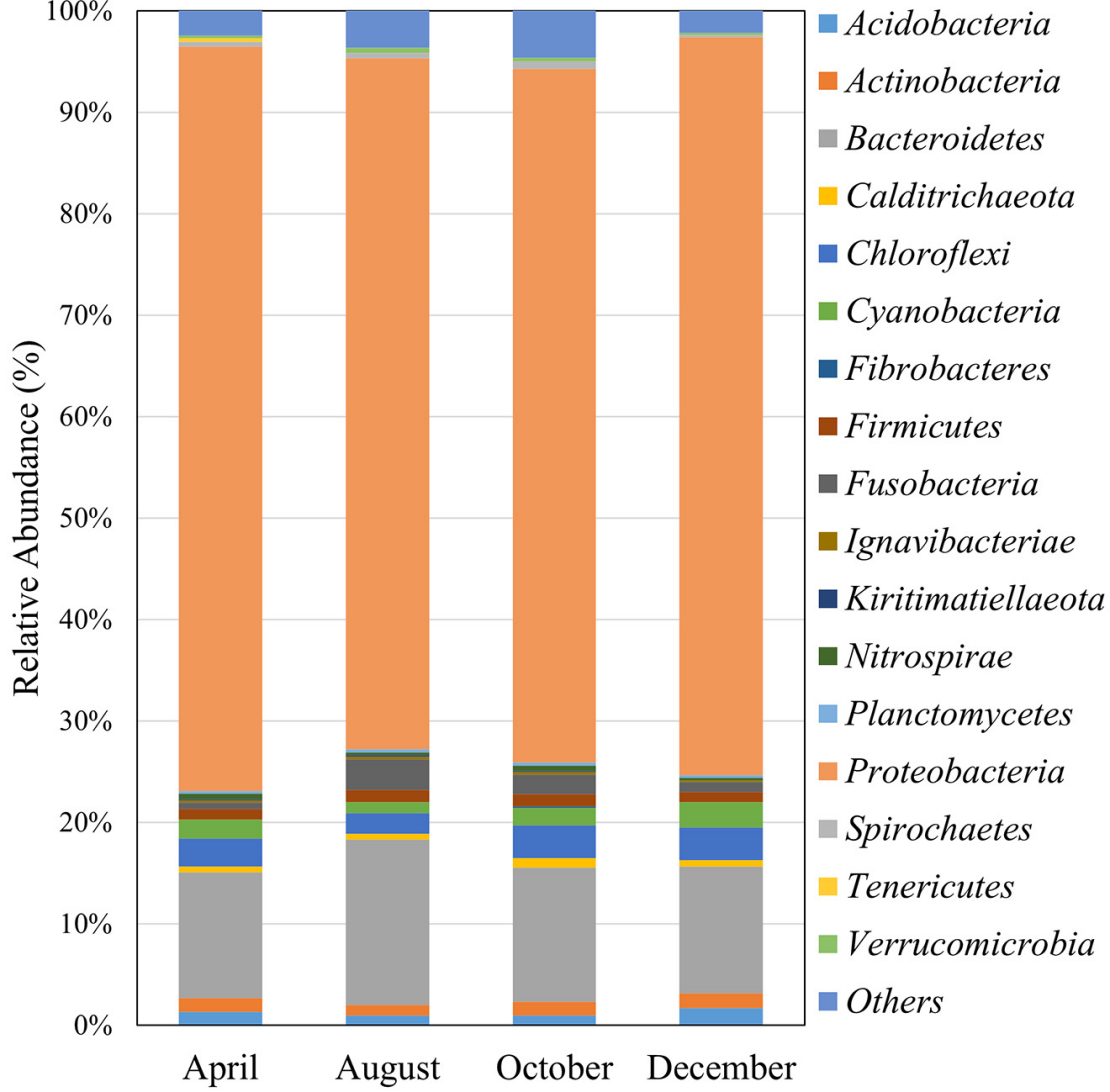
Bar chart of the microbial composition in Jindong Bay in 2019. The figure presents the relative abundance of the main phyla obtained from 16S rRNA gene sequencing. Phyla with relative abundance of less than 0.01% were removed.

The predominant phylum was *Proteobacteria* (relative abundance of 67.91 to 73.34%), followed by *Bacteroidetes* (12.40 to 16.26%), *Chloroflexi* (2.00 to 3.22%), *Cyanobacteria* (1.10 to 2.49%), *Actinobacteria* (1.01 to 1.42%), *Firmicutes* (0.99 to 1.19%), *Acidobacteria* (0.96 to 1.71%), *Fusobacteria* (0.64 to 3.02%), *Calditrichaeota* (0.59 to 0.93%), *Planctomycetes* (0.27 to 0.34%), *Spirochaetes* (0.23 to 0.70%), *Nitrospirae* (0.23 to 0.55%), *Verrucomicrobia* (0.20 to 0.49%), *Ignavibacteriae* (0.17 to 0.26%), *Tenericutes* (0.00 to 0.36%), *Kiritimatiellaeota* (0.00 to 0.0.18%), and *Fibrobacteres* (0.00 to 0.11%), as shown in Fig. 1.

The 16S rRNA gene amplicon sequencing of samples from Jindong Bay reported here is the first result to represent the microbial structure of sediments in the ascidian Styela clava farm. This result will provide valuable resources for future research with respect to Styela clava aquaculture.

### Data availability.

The 16S rRNA gene amplicon sequences from this study are available in the NCBI Sequence Read Archive (SRA) under the accession number PRJNA733697.
